# Talectomy for Equinovarus Deformity in Family Members with Hereditary Motor and Sensory Neuropathy Type I

**DOI:** 10.1155/2014/643480

**Published:** 2014-12-31

**Authors:** Hristo Georgiev, Georgi P. Georgiev

**Affiliations:** University Hospital of Orthopaedics “Prof. B. Boychev”, Medical University Sofia, 56 Nicola Petkov Boulevard, 1614 Sofia, Bulgaria

## Abstract

The treatment of severe rigid neurogenic clubfoot deformities still remains a challenging problem in modern paediatric orthopaedics. In those cases, in spite of being a palliative procedure, talectomy has been advocated for the correction of the deformity thus providing a stable plantigrade foot which allows pain-free walking with standard footwear. Herein, we present the results after talectomy in two patients (brother and sister) affected by a hereditary motor and sensory neuropathy type I, with rigid severe pes equinovarus deformities.

## 1. Introduction

Hereditary motor and sensory neuropathy (HMSN) also known as Charcot-Marie-Tooth disease is the most common inherited neuromuscular disease which includes a group of clinically and genetically heterogeneous disorders of the peripheral nervous system [1]. This neuromuscular disease usually occurs during the first or second decade of life and is characterized by wasting and weakness starting in the legs and spreading to the upper extremities, distal sensory loss, foot deformities, steppage gait, and decrease or absence of tendon reflexes [2, 3]. Foot deformities are commonly present in children with HMSN. The aim of the orthopaedic treatment is correction of the deformity and restoration of the walking ability, when compromised, in order to have a general improvement of quality of life.

In this report, we describe two young patients affected by a HMSN type I, with rigid severe pes equinovarus (PEV) deformities that underwent single-stage corrective surgery.

## 2. Case Report

Herein, we present two patients, brother and sister, 10 and 12 years old, respectively, with a HMSN type I, that underwent talectomy due to untreated severe rigid PEV.

Both patients were born from normal pregnancies. In the female patient birth was performed by vacuum extraction and pneumonia was diagnosed on the tenth day of life. The girl and boy started to walk at 1.4 and 1.2 years, respectively, with a peculiar gait. At 7 and 6 years, respectively, the girl and boy started to fall frequently during walking. Later, in both patients progressive deterioration occurred and they had unstable posture and difficulties in walking and required walking aids. Moderate muscular atrophy and weakness of the distal lower limb muscles were also established. They had family members affected by HMSN type I: the mother's sister and the maternal grandmother's sister.

In both patients, vibratory sensation was absent in the toes; the median and tibial motor nerve conduction velocities were low, with a markedly prolonged distal latency and low amplitudes; no nerve action potentials were elicited from stimulation of the sural nerves; clinical examination revealed that muscle stretch reflexes were absent in the lower limbs and were decreased in the upper limbs; haematological and biochemical screenings were negative.

Based on the neurological examinations and nerve conduction studies a HMSN type I was diagnosed in these patients.

**Figure 1:**
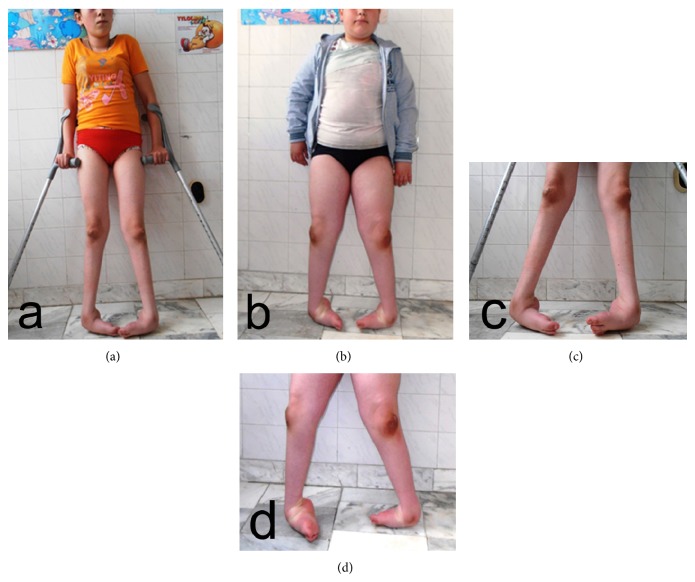
Preoperative photograph of the patients: (a, c) sister and (b, d) brother with hereditary motor and sensory neuropathy type I.

In both patients bilateral rigid PEV deformity was present in a similar pattern. Bean-shaped foot, prominence of the head of the talus, dorsal callosities on the foot, callosities on the knees, medial plantar cleft, deep posterior cleft, absence of normal creases over the insertion of the Achilles tendon, and calcaneal tuberosity situated at a higher level were established. The entire feet were in severe equinus and varus positions with the forefeet adducted (Figures 1(a)–1(d)). After establishing the equinus deviation in the sagittal plane, varus deviation in the frontal plane, derotation of the calcaneopedal block in the horizontal plane, and adduction of the forefoot relative to the hindfoot in the horizontal plane we assessed the PEV deformity as grade IV (both patients/bilateral/score of 17/20 points) according to classification of Diméglio et al. [4].

**Figure 2:**
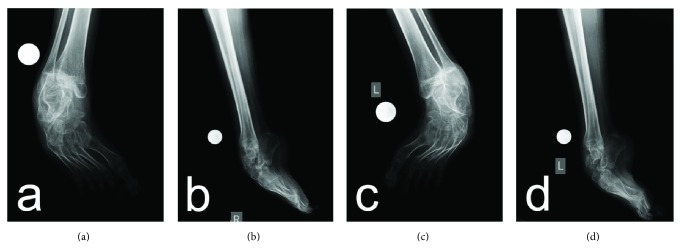
Preoperative anteroposterior (a, c) and lateral (b, d) radiographs of the patients with hereditary motor and sensory neuropathy type I.

**Figure 3:**
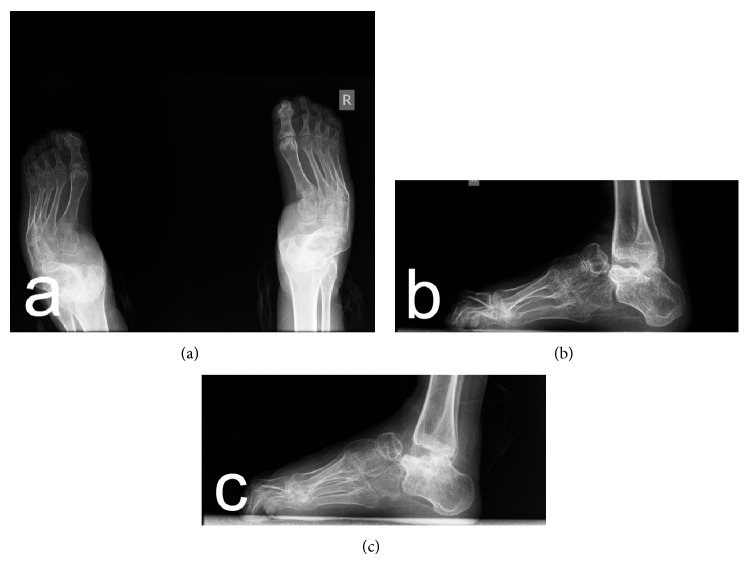
Postoperative anteroposterior (a) and lateral (b, c) radiographs of the patient (sister) with hereditary motor and sensory neuropathy type I.

**Figure 4:**
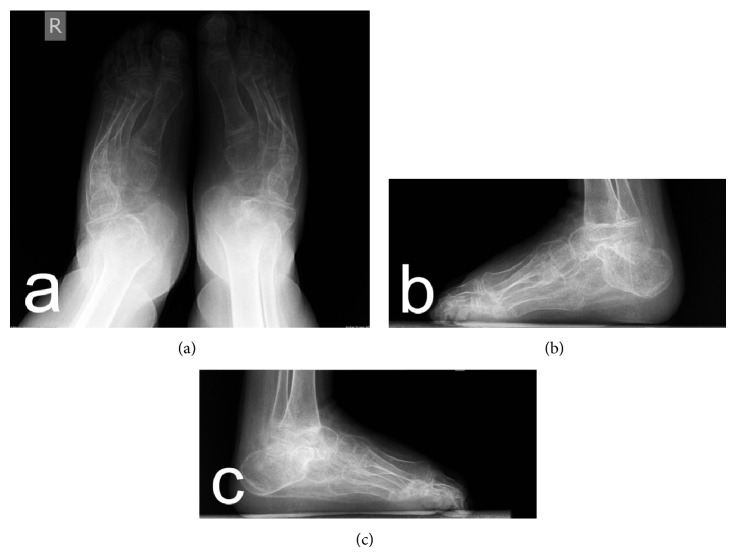
Postoperative anteroposterior (a) and lateral (b, c) radiographs of the patient (brother) with hereditary motor and sensory neuropathy type I.

Preoperative radiographs revealed severe clubfoot deformity with marked degenerative changes in the ankle, subtalar, and talonavicular joints (Figures 2(a)–2(d)). Therefore a decision was made to remove the talus as a single-stage procedure for deformity correction and for improving walking ability which is crucial especially for patients with neuropathic clubfoot deformity. The radiographic changes were confirmed during surgery; the talus was dislocated anteromedially at the ankle joint, with obvious deformation; the neck of the talus was shortened and curved medially and plantarward; there was also visible cartilage degeneration. In the patients with bilateral deformity, the operation of the second foot was performed after cast removal and extraction of K-wires. The patients were evaluated by anteroposterior and lateral radiographs, which revealed satisfactory correction of the deformity (Figures 3(a)–3(c) and Figures 4(a)–4(c)).

**Figure 5:**
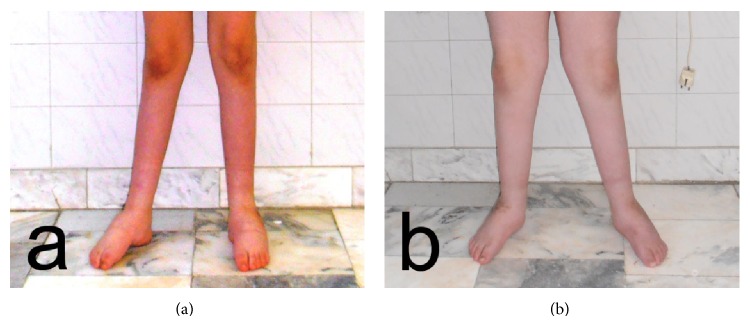
Postoperative photograph of the patients: (a) sister and (b) brother with hereditary motor and sensory neuropathy type I.

In both patients stable plantigrade feet were achieved (Figures 5(a) and 5(b)). The correction of the deformity allowed wearing of shoes, pain-free walking, and no need of walking aids.

Both patients were assessed preoperatively and postoperatively both clinically and radiologically by anteroposterior and lateral radiographs. The mean follow-up was 18 months (±15.3).

### 2.1. Surgical Technique

**Figure 6:**
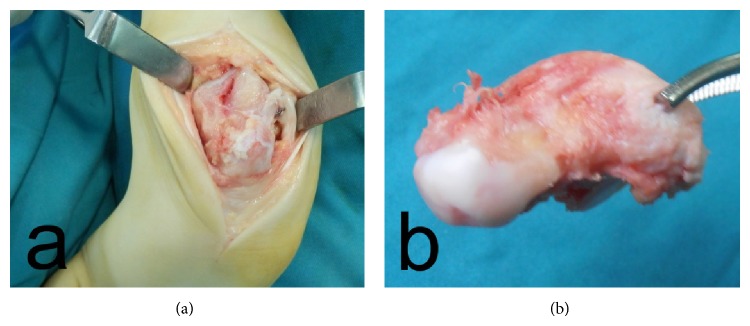
Intraoperative photograph (a) and photograph showing severe degenerative changes of the talus articular cartilage (b).

Surgery is performed through an anterolateral approach to the talus, between the extensor digitorum longus muscle and the peroneus tertius muscle. The talus is visualized after inversion and plantar flexion of the forefoot (Figure 6(a)). After division of the deltoid ligament, anterior and posterior talofibular ligaments, talonavicular ligament, and talocalcaneal ligaments, excision of the talus is performed (Figure 6(b)). Complete excision of the talus is mandatory because the remnants of cartilage could result in late deformity. Correction of the equinus deformity is done by open or percutaneous lengthening of Achilles tendon according to the method of Hoke. After that, derotation of the forefoot and translation of the calcaneus posteriorly until the navicular abuts the anterior edge of the tibial plafond is performed. The foot is aligned perpendicular to the bimalleolar axis of the ankle in 20°–30° external rotation. The foot is fixed in the corrected position with two K-wires through the calcaneus to the distal tibia. A short leg plaster cast is applied for 40 days.

## 3. Discussion

Talectomy, widely used in the past, nowadays has been advocated in children and adolescents as a salvage for the treatment of severe rigid PEV in arthrogryposis or myelomeningocele, as well as in patients with idiopathic PEV in order to provide a stable, plantigrade painless foot, and also in tuberculosis and bone tumors. Triple arthrodesis is also recommended for the treatment of uncorrected clubfeet in older children and adolescents. However, talectomy is preferred in severe, rigid, resistant clubfeet in patients with neuromuscular clubfeet. In these cases stable, plantigrade pain-free foot is seldom achieved even after repeated soft tissue procedures. The patients with severe PEV have unstable posture, weight-bearing on one point, and gait disturbances and require walking aids [5–9].

The advantages of talectomy are the achievement of a stable joint without ankylosis, improved limb function, mechanical support of the limb, and walking without aid after only a single-stage surgery. This salvage procedure provides adequate correction of equinus and varus deformities. The new “tibiocalcaneal joint” is stable and relatively congruent when the foot is in a plantigrade position. The disadvantage of this procedure is the nonphysiological movement in the newly formed joint and the disturbance of normal anatomy [8–10]. The most common complication associated with talectomy is severe arthritis pain [8]. Jóźwiak et al. [11] stated that, despite its palliative character, the talectomy is the method of choice in many cases of severe neurogenic clubfeet.

Menelaus [10] presented good results in 79% of cases (41 feet) after treatment of equinovarus deformity in children with spina bifida and arthrogryposis. Green et al. [12] in the series of 34 feet in 18 children with arthrogryposis showed good results in 71% of cases. The average follow-up in this study was 11 years. The authors recommended salvage for the treatment of PEV deformity, as primary operation or after failure of previous soft tissue corrections. Dias and Stern [6] also achieved excellent and good results after talectomy in 71% of patients with arthrogryposis and myelomeningocele. Cooper and Capello [5] present the longest follow-up after talectomy in patients with poliomyelitis and calcaneovalgus deformity. The authors presented good results in 92% of cases. The average follow-up was 20 years. Günal [13] presented four neurogenic cases with good results after modified technique of talectomy. He made an osteotomy transversely 1 cm proximal to the joint line, about one-third of the width of the tibia perpendicular to the joint line. Thereafter, 1 × 0.5 cm of bone resection was performed and the malleolar fragment was displaced laterally and fixed with a screw. D'Souza et al. [7] presented satisfactory results in 73.7% of cases (14 feet) after treatment of PEV deformity in children with arthrogryposis. The average follow-up in this study was 11.1 years. Legaspi et al. [8] reported the results after talectomy in 15 patients with clubfoot deformity (24 feet: 21 feet in children with arthrogryposis, 2 feet (one patient) with myelomeningocele and 1 foot (one patient) with idiopathic PEV congenitus). Mean follow-up was 20 years. Good results were reported in 33% of cases (8 feet), fair in 42% (10 feet), and poor in 25% (6 feet). Yalçin et al. [14] presented good results in 12 feet and fair in 5 feet after talectomy in patients with various neurological disorders operated on for correction of neglected PEV deformity. The authors recommended this salvage procedure as a limb-sparing procedure not only ensuring a plantigrade foot but also providing proper postoperative orthotic control. Pirpiris et al. [15] compared the results after 31 talectomies (14 isolated talectomies, 17 combined talectomy and calcaneocuboid fusions) for rigid equinovarus deformities predominantly in patients with arthrogryposis. After their retrospective cohort study of 17 children with a mean age at surgery of 5.6 years, the authors conclude that this salvage procedure combined with calcaneocuboid fusion gives better medium-term results. Al-Raggad [9] showed the results after 48 talectomies in 31 children with rigid severe clubfoot deformity (arthrogryposis, 85% of cases; spina bifida, 13%; and one case of idiopathic PEV congenitus), achieving good results in 77% of cases. The average follow-up was 5 years.

Legaspi et al. [8] described for the first time radiographic evidence of tibiocalcaneal arthritis in 8 cases after talectomy. The authors believe that this is due to the longest follow-up in their study. Four of these patients had no pain during walking; in two of them walking was limited by pain, while in the other two patients a fusion between tibia and calcaneus was performed due to severe arthritis pain.

Some authors consider that talectomy alone could not correct the residual forefoot deformity in patients with PEV [6, 16]. This may require additional surgery that should not be considered as a failure of salvage or a poor result.

Some authors recommend posterior translation of the calcaneus. This ensures normal contour of the back of the heel and provides a relative displacement of the “tibiocalcaneal joint” [6, 7, 10]. Legaspi et al. [8] reported a lack of correlation between the clinical outcome and the axis of the “tibiocalcaneal joint.”

## 4. Conclusion

Our results suggest that, with proper indications, talectomy, despite of its palliative nature, has its place in pediatric orthopaedic surgery. This operation may be a method of choice as a “limb-saving procedure” in neurogenic neglected PEV, as well as in adolescents with untreated severe idiopathic congenital PEV in order to obtain a stable plantigrade foot.

## References

[1] Skre H. (1974). Genetic and clinical aspects of Charcot Marie Tooth's disease. *Clinical Genetics*.

[2] Harding A. E., Thomas P. K. (1980). The clinical features of hereditary motor and sensory neuropathy types I and II. *Brain*.

[3] Houlden H., Charlton P., Singh D. (2007). Neurology and orthopaedics. *Journal of Neurology, Neurosurgery & Psychiatry*.

[4] Diméglio A., Bensahel H., Souchet P., Mazeau P., Bonnet F. (1995). Classification of clubfoot. *Journal of Pediatric Orthopaedics Part B*.

[5] Cooper R. R., Capello W. (1985). Talectomy. A long-term follow-up evaluation. *Clinical Orthopaedics and Related Research*.

[6] Dias L. S., Stern L. S. (1987). Talectomy in the treatment of resistant talipes equinovarus deformity in myelomeningocele and arthrogryposis. *Journal of Pediatric Orthopaedics*.

[7] D'Souza H., Aroojis A., Chawara G. S. (1998). Talectomy in arthrogryposis: analysis of results. *Journal of Pediatric Orthopaedics*.

[8] Legaspi J., Li Y. H., Chow W., Leong J. C. Y. (2001). Talectomy in patients with recurrent deformity in club foot. *Journal of Bone and Joint Surgery—Series B*.

[9] Al-Raggad M. (2013). Talectomy in the treatment of resistant talipes equinovarus deformity: the indications and results. *International Journal of Biological and Medical Research*.

[10] Menelaus M. B. (1971). Talectomy for equinovarus deformity in arthrogryposis and spina bifida. *The Journal of Bone and Joint Surgery—Series B*.

[11] Jóźwiak M., Idzior M., Kowalski I. (2007). Talectomy for the clubfeet treatment in children with myelomeningocoele. *Chirurgia NarzadówRruchu i Ortopedia Polska*.

[12] Green A. D. L., Fixsen J. A., Lloyd-Roberts G. C. (1984). Talectomy for arthrogryposis multiplex congenita. *Journal of Bone and Joint Surgery*.

[13] Günal I. (1994). Talectomy for osteoporotic and neuropathic feet. 7 cases followed for 2-3 years. *Acta Orthopaedica Scandinavica*.

[14] Yalçin S., Kocaoğlu B., Berker N., Erol B. (2005). Talectomy for the treatment of neglected pes equinovarus deformity in patients with neuromuscular involvement. *Acta Orthopaedica et Traumatologica Turcica*.

[15] Pirpiris M., Ching D. E., Kuhns C. A., Otsuka N. Y. (2005). Calcaneocuboid fusion in children undergoing talectomy. *Journal of Pediatric Orthopaedics*.

[16] Segal L. S., Mann D. C., Feiwell E., Hoffer M. M. (1989). Equinovarus deformity in arthrogryposis and myelomeningocele: evaluation or primary talectomy. *Foot and Ankle*.

